# *Chlamydia trachomatis* isolated from cervicovaginal samples in Sapporo, Japan, reveals the circulation of genetically diverse strains

**DOI:** 10.1186/s12879-020-4780-y

**Published:** 2020-01-16

**Authors:** Jeewan Thapa, Takanori Watanabe, Mana Isoba, Torahiko Okubo, Kiyotake Abe, Kunihiro Minami, Hiroyuki Yamaguchi

**Affiliations:** 1 Department of Medical Laboratory Science, Faculty of Health Sciences, Hokkaido University, Nishi-5 Kita-12 Jo, Kita-ku, Sapporo, Hokkaido 060-0812 Japan; 2 Toho Obstetrics and Gynecology Hospital, Higashi-15, Kita-17 Jo, Higashi-ku, Sapporo, 065-0017 Japan

**Keywords:** Chlamydia trachomatis, *ompA*, Multilocus sequence analysis, Genotypes

## Abstract

**Background:**

This study was conducted to understand the molecular epidemiology of circulating *Chlamydia trachomatis* (Ct) strains in Sapporo, Japan.

**Methods:**

A total of 713 endocervical samples collected from April 2016 to March 2019 were screened for Ct. The obtained Ct positive samples were analyzed by *ompA* genotyping and multilocus sequence analysis (MLSA).

**Results:**

Eighty-three (11.6%) samples were positive for Ct plasmid DNA. Sequence analysis of the *ompA* gene from the 61 positive cases revealed eight genotypes: F (40.9%), E (19.6%), D (14.7%), G (9.8%), H (6.5%), I (3.2%), K (3.2%), and J (1.6%). The globally dominant genotype E and F strains were highly conserved with 13 *ompA* genetic variants being detected, whereas genotype D strains were the most diverse. Genetic characterization of D strains revealed that D1 genetic variants may be potentially specific to Sapporo. MLSA revealed 13 unique sequence types (STs) including four novel STs from 53 positive samples, with the globally dominant STs 39 and 19 being predominant. STs 39, 34, and 21 were exclusively associated with genotypes E and F indicating their global dominance. Novel ST70 and ST30 were specifically associated with genotype D.

**Conclusion:**

Our study has revealed the circulation of genetically diverse Ct strains in the women population of Sapporo, Japan. We suggest identifying a transmission network of those successful strains and implementing public health prevention strategies to control the spread of Ct in Sapporo.

## Background

*Chlamydia trachomatis* (Ct) is the leading cause of bacterial sexually transmitted infections (STIs) with an estimated 127 million new cases annually worldwide [1, 2], mostly affecting sexually active adults [3]. In Japan, Ct is also a major cause of STIs and cases have been reported across the whole country [4]. Pregnant women in Japan are routinely tested for Ct because of its potential adverse effects during pregnancy [4].

Sequence analysis of the *ompA* gene that encodes a major outer membrane protein (MOMP) has divided Ct strains into three clinically distinct genotypes: trachoma (A, B, C), urogenital infections (D, E, F, G, H, I, J, K), and lymphogranuloma venereum (L1, L2, L3) [5, 6]. Among the urogenital genotypes, E and F are the predominant genotypes, accounting for up to 60–70% of cases [4–10]. The genotypes E and F have been reported to be the globally dominant strains [6, 7, 11] and are suggested to have undergone recent genetic recombination and lineage expansion with few variations in the *ompA* gene [9, 11, 12]. However, this information is mostly derived from European and American studies, so may not accurately reflect the situation in other geographic regions.

Molecular epidemiological information on Ct from urogenital infections is limited in Asia. Previous studies from Japan estimated the overall average prevalence to be 5.8% but revealed a relatively higher prevalence of Ct in Sapporo, for example 14.3% (40/280) from endocervical samples of women in 2010 [4] and 11.3% (218/1917) from endocervical swabs of pregnant women in 1997 [13]. The dominant genotypes from 40 endocervical samples collected in 2010 were D (30%), F (12.5%), and E (7.5) [4], whereas, in 1997, the dominant genotypes from 218 endocervical samples were E (24.3%), D (19.3%), and F (17.9%). In a study in Tokyo [14], the dominant genotypes from 44 clinical STI samples (25 men, 19 women) were E (25%), F (20.5%), G (18.1%), and D (15.9%). The genotypic prevalence of Ct in other countries in Asia, such as China [15], Taiwan [16], Korea [17], and India [18], revealed the broader distribution of genotypes D, E, and F. Since Ct strains have diversified regionally and globally [7], the molecular epidemiology of Ct strains in other geographic regions, such as Japan, may differ via fitness of the strains, cross-host transmission events or examined population.

The discriminatory power of *ompA* genotyping is limited as this gene is under immune selection and represents only 0.1% of the genome [6, 19]. Multilocus sequence analysis (MLSA) of Ct strains along with *ompA* genotyping has higher discriminatory power and is useful in identifying emerging strains [6, 7, 19]. Furthermore, these combined techniques are considered to be suitable for understanding the transmission dynamics of Ct strains [20], and show greater congruency with whole genome analysis [21]. However, the applicability of MLSA of Ct urogenital strains is limited to European and American strains and has not been applied to Japanese or Asian strains, so a detailed picture of Ct molecular dynamics in other geographical regions including Japan is lacking. Thus, the main aim of this study was to determine the genetic characteristics of Ct strains in Sapporo by *ompA* genotyping and MLSA. Such data may provide insight into the transmission dynamics of Ct strains in Sapporo and will contribute to improved control strategies.

## Methods

### Endocervical sample collection

In collaboration with Sapporo Toho Hospital, a community obstetrics and gynecology hospital, we collected endocervical swabs form 713 women who were attending their first prenatal visit or those with clinical manifestations, including bacterial vaginosis, from April 2016 to March 2019. The average age (±SD) of women attending was 28 ± 5.1 years. The samples were collected and processed as previously described [4, 22]. Briefly, the endocervix was scraped with a sterile cotton applicator and the applicator was immediately immersed and resuspended in 5 ml SPG (0.2 M sucrose, 3.8 mM KH_2_PO_4_, 6.7 mM Na_2_HPO_4_, 5 mM l-glutamic acid, pH 7.4) and immediately transported at 4 °C. The sample was divided into 10 tubes (500 μl each), and then stored at ˗80 °C until use.

### DNA extraction

A tube containing 500 μl of SPG solution was used for DNA extraction. The solution was centrifuged at 13,000 g for 5 min and the resulting pellet was used for DNA extraction. The DNA was extracted using a Labopass Tissue Mini kit (Cosmo Genetech, Co., Ltd., Seoul, South Korea), according to the manufacturer’s instructions. The DNA was eluted in 50 μl of the elution buffer, quantified spectrophotometrically using Eppendorf Biophotomoter® D30 and stored at − 20° until use.

### Ct detection by PCR

Initially, the quality of the extracted DNA was confirmed by conventional PCR amplification of the bacterial 16S rDNA gene, which is widely conserved across bacteria [23]. All PCRs were performed using iCycler™ BIO-RAD. Reaction mixture of each amplification assay was prepared to a volume of 20 μl consisting of 8.2 μl of sterile dH_2_O, 10 μl of Quick Taq HS DyeMix (Tyobo Co., Ltd., Japan), 0.3 μM primers, and 1 μl of template DNA (average DNA concentration in each sample 50 ± 45 ng/μl). The PCR cycle for 16S rDNA consisted of an initial denaturation (5 min at 95 °C), followed by 30 cycles of denaturation (30 s at 95 °C), annealing (30 s at 55 °C), and extension (30 s at 72 °C), followed by a final extension (5 min at 72 °C). Positive (Ct D/UW3 Cx) and negative (dH_2_O) controls were used in each amplification assay. Upon amplification, PCR products were separated by 2% agarose gel electrophoresis and visualized by ethidium bromide staining. Thirty-two samples that were negative were omitted. Finally, 681 samples were used to detect Ct by conventional PCR amplification targeting 241 bp of genetically conserved Ct plasmid DNA as previously described [22, 23]. The PCR reaction mixture was prepared similarly to that of 16 s rDNA as described above. The PCR cycle consisted of an initial denaturation (5 min at 95 °C), followed by 40 cycles of denaturation (30 s at 95 °C), annealing (30 s at 60 °C), and extension (30 s at 72 °C), followed by a final extension (5 min at 72 °C).

### *OmpA* genotyping and MLSA analysis

The obtained Ct positive samples were analyzed by *ompA* conventional PCR using the previously described P1 and OMP2 primers [5]. The PCR cycle consisted of an initial denaturation (5 min at 98 °C), followed by 40 cycles of denaturation (10 s at 98 °C), annealing (30 s at 55 °C), and extension (10 min at 72 °C), followed by a final extension (5 min at 72 °C). The amplified product consisted of a fragment of 1100 bp of *ompA* gene. Then, these amplified PCR products of *ompA* were purified using FastGene Gel/PCR Extraction Kit (Nippon Genetics Co., Ltd., Japan) and bidirectionally sequenced using both forward and reverse primers [4, 22]. The quality of raw sequence data was analyzed with FinchTV Version 1.5.0 software, and then the individual consensus *ompA* sequences were built using BioEdit version 7.0.5 software. The obtained sequence was used for genotyping based on BLAST similarity search tool in the National Center for Biotechnology Information (www.ncbi.nlm.nih.gov). The sequences were compared with reference sequences: A/Sa1 (M58938), B/TW-5 (M17342), C/TW3 (M17343), D/B-120 (X62918), E/Bour (X52557), F/IC-Cal3 (X52080), G/UW57 (AF063199), H/Wash (X16007), I/UW-12 (AF063200), J/UW36 (AF063202), K/UW31 (AF063204), L1/440 (M36533), L2/434 (M14738), and L3/404 (X55700) by sequence alignment in BioEdit to identify any nucleotide substitution. *Chlamydia muridarum* MoPn (M64171) was used as an out-group sequence for phylogenetic analysis. MEGA7/X was used to align and construct a phylogenetic tree [24]. From the alignment length 889 bp, a neighbor-joining phylogenetic tree was constructed with maximum composite likelihood nucleotide substitution model, and with a bootstrap value of 1000 replicates.

For MLSA analysis, Ct Multilocus Sequence typing (MLST) *C. trachomatis* scheme developed by Dean et al., 2009 [6] that comprises of seven housekeeping genes, *glyA, mdhC, pdhA, yhbG, pykF, leuS,* and *lysS*, was targeted in this study. Each of the above housekeeping gene fragments of the Ct positive sample was amplified by a conventional PCR. The PCR cycle consisted of an initial denaturation (5 min at 95 °C), followed by 40 cycles of denaturation (30 s at 95 °C), annealing (30 s at 55 °C), and extension (30 s at 68 °C), followed by a final extension (5 min at 68 °C). The PCR products were sequenced using the forward primer and reverse primers, and processed similarly to that of *ompA* gene. The obtained sequences were entered into the Chlamydiales MLST databases (Dean MLST Ct scheme, https://pubmlst.org/bigsdb?db=pubmlst_chlamydiales_seqdef&page=sequenceQuery [25]) to obtain the allelic number of each gene. By combining the allelic profiles of the seven genes, the sequence type (ST) of each sample was determined. Finally, the concatenation of the seven genes was performed in the same order as Dean Ct MLST scheme and used for phylogenetic analysis. MEGA7/X was used to align and construct a phylogenetic tree [24]. The neighbor-joining phylogenetic tree was constructed with a maximum composite likelihood nucleotide substitution model consisting of a bootstrap value of 1000 replicates.

## Results

### Ct detection from clinical samples

From the 713 clinical samples, 681 (95.5%) were positive for 16S rDNA indicating the presence of bacteria in the samples. Further analysis of those 681 samples revealed that 83 (11.6%) samples were positive for Ct plasmid DNA. The average age (±SD) of women who were positive for Ct was 28 ± 5.1 years.

### *OmpA* genotyping

**Fig. 1:**
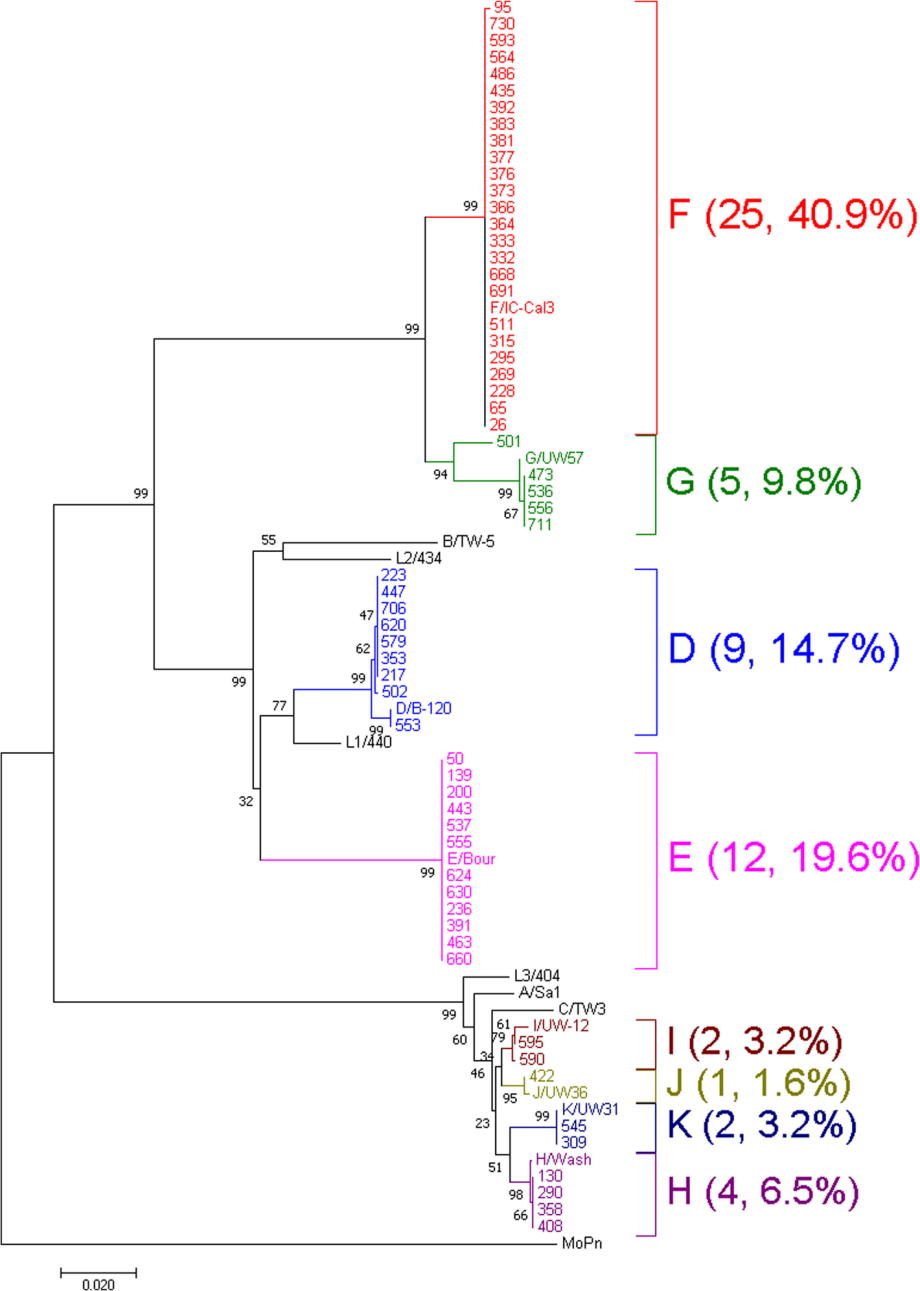
Mid-rooted phylogenetic tree generated by the neighbor-joining method of the *C. trachomatis ompA* nucleotide sequences from 61 clinical strains isolated from Sapporo and 15 reference sequences available from the GenBank database. The sequence of *C. muridarum* was used as an outgroup. The clinical samples and their corresponding genotypes (number of samples, %) are represented by identical colors. The scale bar represents the number of nucleotide substitutions per site

All of the 83 Ct plasmid positive samples were analyzed by *ompA* PCR and 67 samples were positive. However, six samples did not yield quality sequence so were not analyzed further. Thus, we were able to confirm 61 *ompA* genotypes. As Ct prevalence was determined by PCR of 241 bp of the conserved plasmid, the higher number of plasmid copies per sample may have resulted in this greater PCR positive rate. We had confirmed negative PCR results by repeating PCR by increasing concentration (2 μl) and decreasing (0.5 μl) concentration of template DNA. The lower positive rates for *ompA* may be due to the quantity and quality of Ct DNA that was directly extracted from clinical samples [9]. The predominant genotypes were F (40.9%), E (19.6%), and D (14.7%), followed by G (9.8%), H (6.5%), I (3.2%), K (3.2%), and J (1.6%) (Fig. 1).

Sequence analysis divided the Ct strains into 13 genotypic variants (Table 1). All 12 strains of genotype E were conserved, whereas 96% (24 of 25) of strains of genotype F were conserved (Table 1). Genotype D was the most divergent type with three variants. The major D1 variant consisted of seven strains with potentially Sapporo-specific 129C → T, 184G → A, 186 T → G, 195C → T, 636A → T nucleotide substitutions. The mutations 184G → A and 186 T → G resulted in an amino acid change from valine to methionine. Descriptions of the nucleotide substitutions from other genotypes when compared to the reference sequence are presented in Table 1. Representative sequences of the 13 *ompA* genotypic variants were deposited into the GenBank database (accession numbers: LC498598 to LC498610).

**Table 1:** Nucleotide changes in the *ompA* gene of clinical strains of *C. trachomatis* compared with reference sequences

Genotype (no. of strains)		Nucleotide change	Amino acid change	Accession number
D (9)	D1 (7)	(129C → T,	(Synonymous,	LC498598
		**184G → A,**	Val → Met,	
		**186 T → G,**		
		195C → T	Synonymous,	
		636A → T)^a^	Synonymous)^b^	
	D2 (1)	()^a^ + **977C** → **T**^**a**^	()^b^ +Ala→Val	LC498599
	D3 (1)	No mutations		LC498600
E (12)		No mutations		LC498601
F (25)	F1 (24)	No mutations		LC498602
	F2 (1)	397C → A	Arg → Ser	LC498603
G^c^ (6)	**G1 (4)**	**487G** → **A**	Gly → Ser	LC498604
	**G2 (1)**	**487G** → **A** + 23 SNPs	Gly → Ser	LC498605
H (4)		**272A** → **G**	Asn → Ser	LC498606
		850C → T	Synonymous	
I (2)	I1 (1)	684G → A	Synonymous	LC498607
		**764 T → C**	Ile → Thr	
		810C → T	Synonymous	
		**1000 T → G**	Ser → Ala	
		**1007C → G**	Ala→Gly	
		Insertion of codon AGC between 1008 and 1009	Insertion of Gly	
		**1011A → T**	Glu → Val	
		**1017A → C**	Glu → Ala	
	I2^d^ (1)	684G → A	Synonymous	LC498608
		**764 T → C,**	Ile → Thr	
		810C → T,	Synonymous	
		**938C** → **T**	Ala→Val	
J (1)		369C → T	Synonymous	LC498609
K (2)		**293A** → **G**	Asn → Ser	LC498610

Bold letters indicate nonsynonymous mutations

Reference sequences used for comparison with sequences obtained in this study were: D/B-120 (X62918), E/Bour (X52557), F/IC-Cal3 (X52080), G/UW57 (AF063199), H/wash (X16007), I/UW-12 (AF063200), J/UW36 (AF063202), and K/UW31 (AF063204)

()^a^ common mutations [129C➔T, 184G➔A, 186 T➔G, 195C➔T, 636A➔T] found in *C. trachomatis* genotype D strains isolated in Sapporo

()^b^ amino acid changes caused by a cluster of common mutations, denoted by ()^a^ in *C. trachomatis* genotype D strains isolated in Sapporo

^c^One sample of genotype G was confirmed using the forward primer, but substitution 487G➔A could not be confirmed in this sample.

^d^Nucleotide substitutions 1000 T➔G, 1007C➔G, 1011A➔T, and 1017A➔C and insertion of codon AGC between positions 1008 and 1009 were not analyzed in this strain

**Fig. 2:**
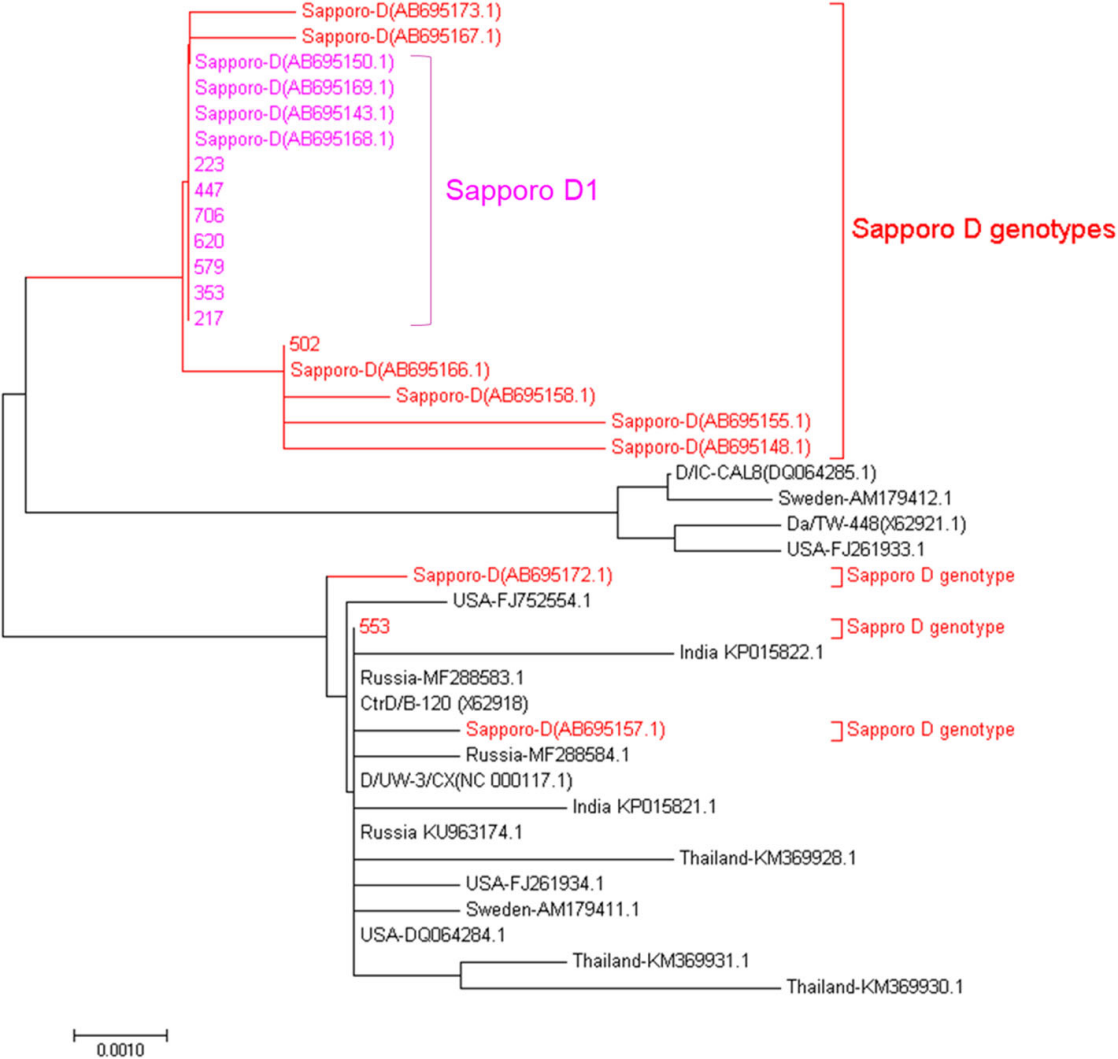
Multilocus sequence analysis (MLSA)-based mid-rooted phylogenetic tree of the concatenated nucleotide sequences of seven MLST loci of 53 *C. trachomatis* strains isolated from Sapporo. Reference sequences were obtained from the Chlamydiales MLST database https://pubmlst.org/bigsdb?db=pubmlst_chlamydiales_seqdef&page=profiles. Each clinical strain is represented by its identification number (*ompA* genotype/collection year). Two red lines divide the tree into two distinct groups: cluster 1 and cluster 2. Sequence types (STs) are shown in colored boxes to represent the corresponding clinical strains. The scale bar represents the number of nucleotide substitutions per site

To confirm whether D1 was a potentially Sapporo specific genetic variant, we analyzed the uncharacterized *ompA* sequences from the previous Sapporo Ct strains collected in 2010 [4], for which genotype D was reported to be the predominant genotype (*n* = 12, 40%). We downloaded all the deposited sequences from GenBank and analyzed those sequence by performing BLAST and comparing with the Ct reference strains as described in materials and methods. Sequence analysis revealed 27 genetic variants, where genotype D was the most diverse with nine genetic variants (see Additional file 1). Furthermore, we downloaded additional global D strains and constructed a neighbor-joining phylogenetic tree with maximum composite likelihood nucleotide substitution model consisting of a bootstrap value of 1000 replicates. The neighbor-phylogenetic analysis of global D strains suggest that D1 strains may be potentially Sapporo specific (Fig. 2).

### MLSA

MLSA of all 83 Ct plasmid positive samples confirmed the STs for 53 samples. A total of 13 unique STs including four novel STs were determined, and previously reported dominant STs, ST39 (22.6%), ST19 (18.8%) [6, 7, 19], and ST21, were of the majority types (Table 2). The allelic profile of four novel STs identified in this study was deposited into pubMLST database https://pubmlst.org/chlamydiales/ and properly assigned with new STs (ST70, ST85, ST86, and ST87). ST19 consisted of diverse *ompA* genotypes (D, G, H, J, K), whereas some STs were specific to some *ompA* genotypes (Table 2).

**Table 2:** Sequence types (STs), allelic profiles, and *ompA* genotypes of *C. trachomatis* strains from Sapporo

ST	*C. trachomatis* MLST alleles							No. of samples (%)	*ompA* genotypes (number)
	*glyA*	*mdhC*	*pdhA*	*yhbG*	*pykF*	*lysS*	*leuS*		
19	3	3	3	6	6	4	3	10 (18.8)	D (3), G (2), H (1), J (1), K (2), NA (1)
21	3	3	3	2	6	4	8	8 (15.9)	E (1), F (7)
23	3	3	3	6	6	8	3	3 (5.7)	H (3)
30	3	3	5	6	6	4	3	4 (7.5)	D (3), NA (1)
34	6	3	3	2	7	4	3	4 (7.5)	F (3), I (1)
39	6	4	3	2	7	4	3	12 (22.6)	E (9), F (2), NA (1)
52	3	3	5	6	6	1	3	4 (7.5)	G (4)
54	6	3	3	2	6	4	3	1 (1.9)	F (1)
81	6	4	8	2	7	4	3	1 (1.9)	E (1)
70 (novel ST)	3	3	8	6	6	4	3	3 (5.7)	D (2), NA (1)
85 (novel ST)	3	3	3	2	7	4	8	1 (1.9)	F (1)
86 (novel ST)	7	3	3	6	6	1	6	1 (1.9)	NA (1)
87 (novel ST)	3	3	3	6	6	4	9	1 (1.9)	I (1)

*NA ompA* genotype not available

**Fig. 3:**
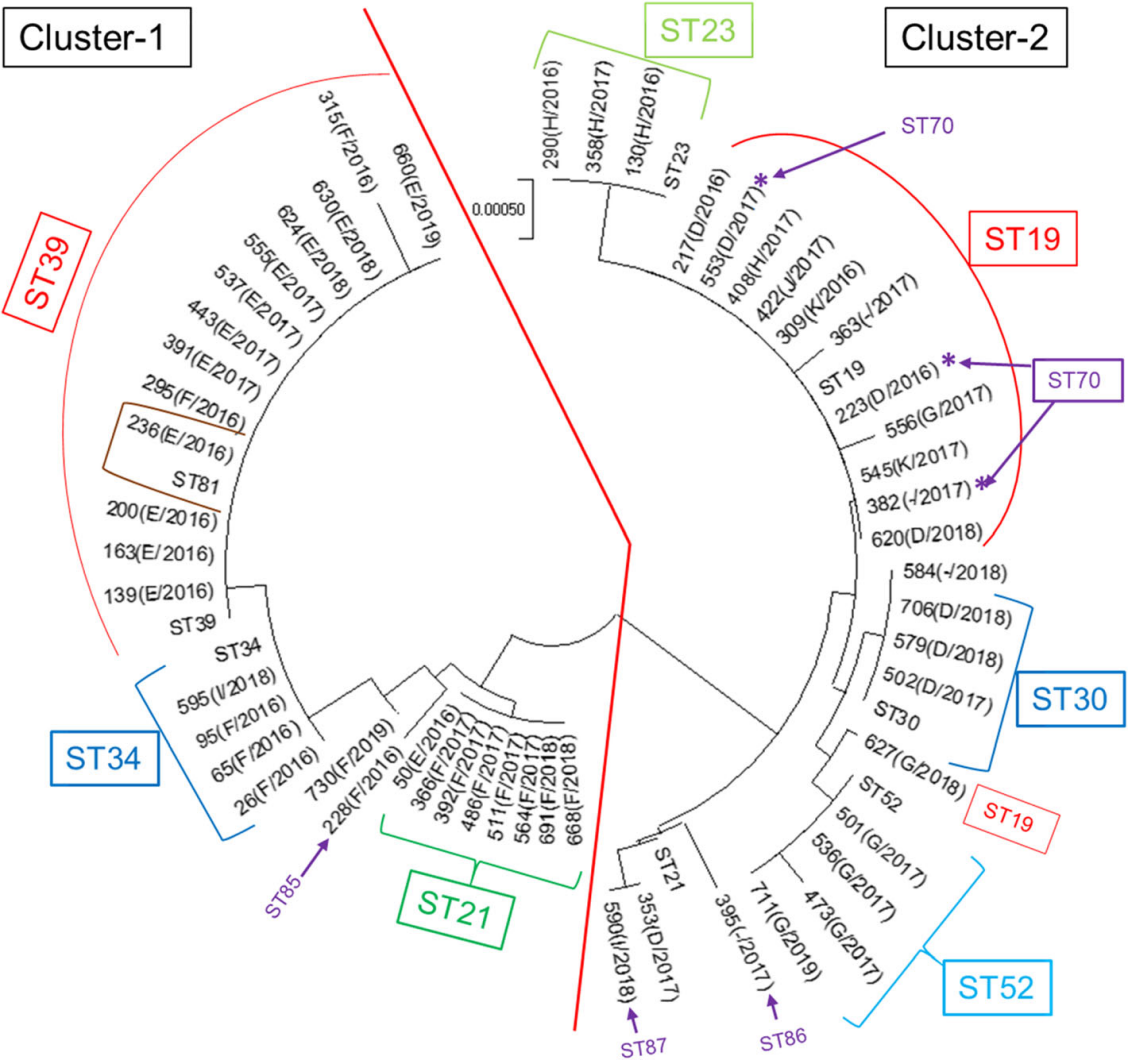
Phylogenetic tree of the *C. trachomatis ompA* D genotype sequences from nine clinical strains isolated in this study, 12 clinical strains from Sapporo from a previous study (4), four reference strains, and other clinical strains from the USA, Russia, Sweden, Thailand, and India. The strains isolated in this study are indicated by their identification number whereas other strains are indicated by their GenBank accession numbers. Strains represented in red indicate overall Sapporo D genotypes, whereas strains represented in fuchsia indicate Sapporo D1 genetic variants. The neighbor-joining method with a bootstrap value of 1000 replicates was used to construct the phylogenetic tree. The scale bar represents the number of nucleotide substitutions per site

MLSA concatenated sequence phylogenetic analysis (Fig. 3) revealed two distinct clusters of samples. Cluster 1 comprised STs 21, 34, 39, 54, 81, and new type-2, and exclusively consisted of globally dominant genotypes E and F. Cluster 2 comprised STs 19, 23, 30, 52, and new types 1, 3, and 4. This cluster consisted of genotypes D, G, H, I, and K, where D had diverse STs (19, 30, and 70).

## Discussion

To our knowledge, this is the first investigation of the genetic characterization of urogenital Ct strains from Japan and Asia by MLSA along with *ompA* genotyping, although the MLST data of trachoma Ct strains are available from Taiwan, Nepal, and Saudi Arabia [6, 7]. In this study, the dominant *ompA* genotype was found to be F (40.9%), followed by E (19.6%) and D (14.7%). This differed from the prevalence of D (30%), F (12.5%), and E (7.5%) genotypes from samples collected from Sapporo in 2010 [4] and E (25%), F (20.5%), and D (15.9%) genotypes from samples collected from Tokyo [15]; thus broadly indicating the distribution of D, E, and F genotypes in Japan and other Asian countries [15–18].

The predominance of genotypes E and F was broadly similar to previous reports from Europe and America [5–11, 21]. All strains of E and F type had similar *ompA* sequences (Fig. 1, Table 1) indicating their success in dealing with the human immune system and becoming established as globally dominant strains [11, 21, 26].

We found that genotype D was the most genetically diverse with three genetic variants, with variant D1 being detected in seven strains (Table 1, Fig. 1). The dominant D1 genotype had Sapporo specific 129C → T, 184G → A, 186 T → G, 195C → T, and 636A → T nucleotide substitutions, with the 184G → A and 186 T → G nucleotide substitutions leading to an amino acid change from valine to methionine. To the best of our knowledge, the D1-specific nucleotide substitution described above has not previously been reported in other strains outside of Sapporo. Phylogenetic analysis of D strains from Sapporo and the world also showed that Sapporo D1 strains may be potentially specific to Sapporo (Fig. 2). We therefore hypothesize that D1 strain may have selectively evolved and adapted within the geographical region of Sapporo.

Two G variants were detected in this study, of which G1 harbored the *ompA* nucleotide substitution 487G → A (Gly → Ser) as previously reported from Russia and Sweden [5, 9, 27] (Table 1). All of the H genotypes shared similar mutations (272A → G, 850A → T) as previously reported from Russia [27] (Table 1). The J and K variants were similar to those reported from Taiwan [16], thus suggesting that the distribution of the reported G, H, J, and K genetic variants may not be limited to Sapporo.

From MLSA analysis, the predominant sequence type was found to be ST39 with 12 strains consisting of nine E genotypes, two F genotypes, and one strain with an untyped genotype. ST39 is a common ST found in Europe and America and is mostly associated with the successful E genotype [6, 7, 18]. The second most prevalent ST was ST19, which was detected in 10 strains of different genotypes (3D, 2G, 1H, 2 K, and 2 not *ompA* genotyped) as reported in previous studies [6, 7, 18]. The third most prevalent ST was ST21, which was detected in seven strains of F genotype and was associated with cluster 1. However, the reference sequence of ST21 of G genotype was associated with cluster 2 and the reason for this discrepancy was nucleotide substitutions (1425G → A, 1489C → T, 1491A → G, 1494C → T, 1509C → T, and 1573G → A) in the *yhbG* gene of Sapporo Ct strains. Another commonly reported ST34 was mostly associated with F genotype as previously described [6, 7, 18].

In our study, four novel STs were detected comprising ST70 that consisted of two strains of D1 variant and one strain whose genotype was not determined because the sequencing data were invalid. This ST may be specific to D genotypes that are locally adapted in Sapporo (Fig. 3). In this study, ST30 was specifically associated with three strains of D genotype. This ST has previously only been reported in one strain of genotype G [6, 7, 18], so the detection of three strains of ST30 with genotype D supports our hypothesis of the local adaption of reported D strains in Sapporo. ST52 was associated with G1 genotype (Table 2, Fig. 3) as reported in previous studies [5, 7, 9, 18, 26], and ST23 was associated with H genotype as previously reported [9]. The classification of Sapporo strains into two distinct clusters with cluster 1 comprising strains of globally dominant genotypes E and F and cluster 2 comprising other genotypes including genotype D, further supports the circulation of globally dominant and locally adapted Ct strains in Sapporo. However, in-depth analysis of these strains by whole genome sequencing and comprehensive analysis of other Ct isolates from different regions of Japan and other Asian countries will further clarify the molecular dynamics of Ct strains in this region.

## Conclusions

Our study has revealed the circulation of genetically diverse Ct strains in the women population of Sapporo, Japan. From the *ompA* genotyping and MLSA analysis of Sapporo Ct strains, we detected two distinct groups of globally dominant and locally adapted strains circulating in Sapporo. The first group consisted of globally dominant E and F genotypes with globally dominant STs 39 and 34. We identified potentially Sapporo specific D genotypes with STs 19, 30, and 70 circulating in Sapporo. We suggest identifying a transmission network of genotypes E and F of STs 39 and 34, and genotype D of STs 19, 30, and 70, and implementing public health prevention strategies to control the spread of these successful clones in Sapporo.

## Supplementary information

**Additional file 1 : Table S1.** Retrospective analysis of nucleotide changes found in *C. trachomatis ompA* genotypes from a previous study in Sapporo [4] compared with reference sequences.

## Data Availability

The datasets used and/ or analysed during the current study are available from the corresponding author on reasonable request.

## Abbreviations

Ct: *Chlamydia trachomatis*
MLSA: Multilocus sequence analysis
MLST: Multilocus sequence typing
MOMP: Major outer membrane protein
ST: Sequence types
STI: Sexually transmitted infections
