# Supplementary material for: Chlamydia trachomatis isolated from cervicovaginal samples in Sapporo, Japan, reveals the circulation of genetically diverse strains
Source: BMC Infect Dis. 2020 Jan 16;20:53. doi: 10.1186/s12879-020-4780-y (PMC6966806; doi:10.1186/s12879-020-4780-y)
Supplement: Supplementary file 1 — Additional file 1 : Table S1. Retrospective analysis of nucleotide changes found in C. trachomatis ompA genotypes from a previous study in Sapporo [4] compared with reference sequences. [file 12879_2020_4780_MOESM1_ESM.docx]

**Table S1.** Retrospective analysis of nucleotide changes found in *C. trachomatis* *ompA* genotypes from a previous study in Sapporo [4] compared with reference sequences

| **Genotype (total samples)** | **Nucleotide change** | **Amino acid change** | **No of isolates (%)** |
| --- | --- | --- | --- |
| D (12) | (129C🡪T  **184G🡪A, 186T🡪G**  195C🡪T  636A🡪T)^a^ | (Synonymous,  Val🡪Met,  Silent  Synonymous)^b^ | 4 (33.3) |
|  | ( )^a^  + **977C🡪T^a^** | ( )^b^  +Ala🡪Val | 1 (8.3) |
|  | ( )^a^  + 963A🡪C | ( )^b^  + Synonymous | 1 (8.3) |
|  | ( )^a^  + 921A🡪C | ( )^b^  + Synonymous | 1 (8.3) |
|  | ( )^a^  + **977C🡪T^a^**  **669A🡪C** | ( )^b^  +Ala🡪Val  Glu🡪Asp | 1 (8.3) |
|  | ( )^a^  + **977C🡪T^a^**  **452C🡪G**  **455C🡪G**  950C🡪G | ( )^b^  +Ala🡪Val  Ala🡪Gly  Ser🡪Cys  Synonymous | 1 (8.3) |
|  | ( )^a^  + **977C🡪T^a^**  **446A🡪G**  **449C🡪G**  759A🡪T | ( )^b^  +Ala🡪Val  Asn🡪Ser  Ser🡪Cys  Synonymous | 1 (8.3) |
|  | **977C🡪T** | Ala🡪Val | 1 (8.3) |
|  | 488A🡪C | Synonymous | 1 (8.3) |
| E (3) | No mutations |  | 2 |
|  | 934G🡪A |  | 1 |
| F (5) | **672A🡪C**  **675A🡪C** | Glu🡪Asp  Leu🡪Phe | 1 (20) |
|  | 951T🡪A | Ile🡪Asn | 1 (20) |
|  | 1049T🡪G  1050G🡪A  1052A🡪C | Leu🡪Ter  Asn🡪Thr | 1 (20) |
|  | **672A🡪C**  **675A🡪C**  846C🡪A  **961A🡪T** | Glu🡪Asp  Leu🡪Phe  Synonymous Asn🡪Tyr | 1 (20) |
|  | No mutations |  | 1 (20) |
| G (2) | **487G🡪A** | Gly🡪Ser | 2 (100) |
| I^c^ (5) | **278T🡪G**  **465T🡪G**  **466A🡪G**  684G🡪A  **764T🡪C**  840T🡪G | Val🡪Gly  Phe🡪Leu  Asn🡪Asp  Synonymous Ile🡪Thr  Synonymous | 1 (20) |
|  | **466A🡪G**  684G🡪A  **764T🡪C**  810C🡪T | Asn🡪Asp  Synonymous Ile🡪Thr  Synonymous | 1 (20) |
|  | 684G🡪A  **764T🡪C**  810C🡪T | Synonymous Ile🡪Thr  Synonymous | 2 (40) |
|  | 684G🡪A  **764T🡪C**  840C🡪G | Synonymous Ile🡪Thr  Synonymous | 1 (20) |
| J (2) | 369C🡪T | Synonymous | 1 (50) |
|  | No mutations |  | 1 (50) |
| K (4) | **293A🡪G**  456C🡪G  **458C🡪G** | Asn🡪Ser  Synonymous Ala🡪Gly | 1 (25) |
|  | **293A🡪G**  675A🡪C  **677A🡪C**  **835G🡪T** | Asn🡪Ser  Synonymous Glu🡪Ala  Ala🡪ser | 1 (25) |
|  | **293A🡪G** | Asn🡪Ser | 1 (25) |
|  | 1044T🡪C^d^ | Synonymous | 1 (25) |

Bold letters indicate nonsynonymous mutations.

Reference sequences used for comparison with sequences obtained in this study were: D/B-120 (X62918), E/Bour (X52557), F/IC-Cal3 (X52080), G/UW57 (AF063199), H/Wash (X16007), I/UW-12 (AF063200), J/UW36 (AF063202), and K/UW31 (AF063204).

( )^a^ common mutations [129C🡪T, 184G🡪A, 186T🡪G, 195C🡪T, 636A🡪T]

found in *C. trachomatis* genotype D strains isolated in Sapporo.

( )^b^ amino acid changes caused by a cluster of common mutations, denoted by ( )^a^ in *C. trachomatis* genotype D strains isolated in Sapporo.

^c^All five isolates had additional mutations (1000T🡪G, 1007C🡪G, 1011A🡪T, 1017A🡪C) and insertion of codon AGC between positions 1008 and 1009. These positions were not analyzed in the samples isolated in this study.

^d^1044T🡪C was not examined in *C. trachomatis* K samples in this study.
